# Long noncoding RNAs: glycolysis regulators in gynaecologic cancers

**DOI:** 10.1186/s12935-023-02849-2

**Published:** 2023-01-13

**Authors:** Nengyuan Lv, Siyi Shen, Qianying Chen, Jinyi Tong

**Affiliations:** 1 Department of the Fourth School of Clinical Medicine, Zhejiang Chinese Medical University, Hangzhou, 310053 Zhejiang Province People’s Republic of China; 2 Department of Obstetrics and Gynecology, Affiliated Hangzhou First People’s Hospital, Zhejiang University of Medicine, Hangzhou, 310006 Zhejiang Province People’s Republic of China

**Keywords:** lncRNAs, Glycolysis, Gynecologic cancers, Markers

## Abstract

The three most common gynaecologic cancers that seriously threaten female lives and health are ovarian cancer, cervical cancer, and endometrial cancer. Glycolysis plays a vital role in gynaecologic cancers. Several long noncoding RNAs (lncRNAs) are known to function as oncogenic molecules. LncRNAs impact downstream target genes by acting as ceRNAs, guides, scaffolds, decoys, or signalling molecules. However, the role of glycolysis-related lncRNAs in regulating gynaecologic cancers remains poorly understood. In this review, we emphasize the functional roles of many lncRNAs that have been found to promote glycolysis in gynaecologic cancers and discuss reasonable strategies for future research.

## Introduction

The three most common gynaecologic cancers that seriously threaten female lives and health are ovarian cancer (OC), cervical cancer (CC), and endometrial cancer (EC). CC is associated with HPV infection [1]. One study found that from 1990 to 2019, the net drift in CC mortality was − 0.19% per year (95% CI, − 0.46% to 0.08%) in China [2]. These results were mainly due to improved medical standards, early detection, early treatment, and early diagnosis.

EC is associated with cumulative obesity, oestrogen-related exposure, and other reproductive factors [3]. Between 1990 and 2019, the estimated average percentage change in age-standardized mortality rates in EC was 0.85% (95% CI, 0.93 to 0.76) globally [4]. Compared to the traditional dualistic histopathologic classification, the new molecular classification based on genetic risk factors includes four prognostic categories: microsatellite instability hypermutation, tumours with low and high copy numbers, and POLE ultra-mutation [5]. A longer progression-free survival is correlated with the POLE ultra-mutation, which also has the best prognosis [6]. By using this new classification system, patients can receive more personalized care [7].

The factors that influence OC development are not clear. One study found that an imbalanced vaginal microbiome could increase a person's risk of developing OC [8]. However, advanced gynaecologic cancers continue to pose a serious threat to the lives and health of women worldwide. In particular, most cases of OC are diagnosed at an advanced stage, with 50–70% recurring within two years of initial treatment and a 5-year survival rate of less than 30% [9]. R0-targeted primary cytoreductive surgery and platinum-based combination chemotherapy remain the standard first-line treatments for primary OC. In the primary treatment of OC, poly (ADP-ribose) polymerase (PARP) inhibitors and antiangiogenic bevacizumab are increasingly being used as first-line maintenance therapies [10, 11]. According to research by Hanley, opportunistic salpingectomy can reduce OC risk [12], which leads to a primary prevention opportunity for the general population.

The link between aerobic glycolysis and cancer has existed for decades since the “Warburg effect” was proposed [13–16]. Numerous studies have demonstrated that glycolysis is promoted in gynaecologic cancers [17–19]. The pentose phosphate pathway (PPP), glycolytic enzymes, and glucose transporters (GLUTs) are essential components of glycolysis. Moreover, hypoxia-inducible factor-1α (HIF-1α) is a vital regulator [20, 21]. However, the mechanism of action of ideal drugs targeting the glycolytic pathway of gynaecologic cancers is still not clear; therefore, the in-depth study of their molecular mechanism is of great significance.

LncRNAs are a family of nonprotein-coding RNAs over 200 nt in length [22]. In the fields of chromatin dynamics, gene expression, development, differentiation, and protein stability, lncRNAs have been demonstrated to play a regulatory role [23, 24]. In recent years, lncRNAs have been reported to regulate the energy metabolism of tumours and thus affect the malignant behaviour of tumours, which also partially reveals the molecular mechanism of glycolysis reprogramming. This is true for oesophageal cancer [25], breast cancer [26], pancreatic cancer [27] and gynaecologic cancers [22, 28, 29]. LncRNAs can be considered potential indicators of prognosis, diagnosis, and therapeutic targets [30]. However, the in-depth relationship between lncRNAs and glycolysis remains unclear.

This review will provide a brief overview of the known functions of glycolysis and summarize how lncRNAs regulate glucose metabolism through the regulation of glucose transporters and glycolytic genes. Moreover, we emphasize the functional roles of several lncRNAs that have been found to promote glycolysis in gynaecologic cancers and suggest reasonable strategies for future research.

## Glycolysis in gynaecologic cancers

Under normal circumstances, mitochondrial oxidative phosphorylation (OXPHOS) is the main source of adenosine triphosphate (ATP). Notably, there is often more glycolysis in tumour cells, which can only produce two molecules of ATP, even under adequate oxygen conditions.

### ATP rate

Glycolysis can generate ATP 100 times faster than OXPHOS [31]. This results in cancer cells consuming more glucose molecules than normal cells in a defined period. Pfeiffer et al. hypothesized that this ATP production method allows gynaecologic cancer cells to maintain a higher rate of proliferation and resist cell mortality signals [32]. This concept is currently being investigated in the clinical diagnosis of cancers, including gynaecologic cancers, through positron emission tomography (PET) [33].

### PPP

The PPP, which produces nicotinamide adenine dinucleotide (NADPH) and ribose-5-phosphate, is aided by the accumulation of glycolytic intermediates. Both NADPH and ribose-5-phosphate are required for lipid and nucleic acid production. Ultimately, NADPH generation allows gynaecologic cancer cells to retain sufficient amounts of glutathione (GSH), an important nonenzymatic antioxidant. GSH protects cancer cells from antineoplastic drugs by preserving redox balance and counteracting some of the side effects of chemotherapeutic medicines [34–36]. Transketolase (TKTL1) is one of the numerous enzymes involved in the PPP that has attracted attention due to its role in cell survival under stress or starvation [37, 38]. Other evidence suggests that TKTL1 influences cancer cell therapeutic efficacy for medicines such as imatinib [39] and cetuximab [40]. Jin et al. showed that Mycoepoxydiene, a natural substance derived from a marine fungus, inhibited the PPP and thus decreased HeLa cell proliferation [41].

### Glycolytic enzymes

#### HK

Hexokinase (HK) phosphorylates glucose to glucose-6phosphate (G6P) in the first stage of glycolysis, a rate-limiting process that offers direct feedback inhibition and prevents the consumption of cellular ATP [42]. Furthermore, due to its low km for glucose, this enzyme aids in the initiation of glycolysis, especially when serum glucose levels are low. Apart from its glucose OXPHOS action, a form of HK called HKII can attach to voltage-dependent anion channels (VDACs) located on the outer mitochondrial membrane [43, 44]. To ensure that tumour cells entrap adequate glucose, this VDAC-HK interaction inhibits the negative feedback regulation of G6P. The VDAC-HK interaction also gives HK direct access to ATP produced by mitochondria and prevents BAX that binds to VDAC in normal cells from attaching to VDAC [44, 45]. This VDAC-HKII interaction also impacts BAX’s proapoptotic actions in normal cells, where cytochrome C escapes from the mitochondria, causing apoptosis and preventing tumour cell death [46]. In several investigations, HKII has been shown to be useful in predicting therapy responses. Overexpression of this gene, for example, has been linked to chemoresistance in gynaecologic cancers [47].

Lonidamine, the glucose analogue 2-deoxyglucose (2-DG), 3-bromopyruvate (3-BrPA), and others are HKII inhibitors [48]. In two-phase III randomized trials, lonidamine failed to demonstrate any benefit [49]. In a similar fashion, co-administration of 2-DG with other anticancer drugs demonstrated potential anticancer benefits in preclinical models [50]. However, it has been observed that 2-DG has minor effects as a single agent for antiglycolytic therapy, owing to the drug's non-selectivity, which causes damage to normal cells in prostate cancer and intracranial neoplasms [51]. Novel 2-DG analogues, such as WP1122 and others, have reignited interest in glycolysis inhibition as a cancer-fighting technique. When combined with other effective cytotoxic drugs, they might destroy cancer cells synergistically [52]. 3-BrPA has been demonstrated to prevent HKII from attaching to the mitochondrion as well as cause cell death by inhibiting cell cycle advancement and depleting ATP. It reduces the activity of ABC transporters and drug efflux, resulting in increased drug retention. These findings support 3-BrPA's capacity to overcome chemoresistance and enhance cancer therapies [18].

#### PFK

Although it is difficult to directly suppress phosphofructokinase (PFK) since it is required for glycolysis in normal cells, it may be possible to target it indirectly. By activating fructose-2,6-bisphosphate (F2,6BP), PFKFB3 indirectly activates PFK in cancer [53]. 3-(3-pyridinyl)-1-(4-pyridinyl)-2-propen-1-one (3PO) is a new small drug based on dipyridinyl-propenone that inhibits G2-M phase cell cycle progression by lowering intracellular F2,6BP levels, glucose uptake, and lactate generation [54, 55]. In mice with leukaemia, lung, and breast adenocarcinoma xenografts, 3PO treatment inhibited tumour development in vivo [56]. Furthermore, 3PO might improve the cytotoxic impact on OC cells when combined with cisplatin or paclitaxel [57].

#### PKM2

Pyruvate kinase muscle isozyme 2 (PKM2) catalyses the last glycolytic step, converting phosphoenolpyruvate to pyruvate, and is a rate-limiting enzyme [58]. PKM2 has several distinguishing characteristics [59]. First, the PKM2 protein in cancer tissues switches to a low-activity dimer form. Because dimeric PKM2 has low catalytic activity, it increases the formation of glycolytic intermediates by activating other PPP enzymes and glycerol synthesis, as well as creating NADPH. This inhibits the creation of reactive oxygen species (ROS), generates the materials required for fast cell growth, and increases chemotherapy resistance in cancer cells [60–62]. Second, cell invasion and metastasis are promoted by the relocation of PKM2 in the nucleus [63–65]. PKM2 regulates glycolysis and is linked to poor prognosis, chemoresistance, cell migration, and invasion in gynaecologic cancers [66–69]. A PKM2 inhibitor dramatically slowed the glycolytic rate and lowered the extracellular acidification rate in an OC cell line in a previous study [59]. MHY2245, a novel SIRT1 inhibitor, promotes autophagy and reduces energy consumption in OC cells via the PKM2/Mammalian target of rapamycin (mTOR) pathway [70]. Compound 3 K, another inhibitor, causes autophagic cell death in OC cells by interrupting glycolysis [71].

#### LDHA

Lactate dehydrogenase A (LDHA) catalyses the conversion of pyruvate to lactate in hypoxic cells. Lactate build up lowers intracellular pH, which is detrimental to the cell [72, 73]. Evidence shows that LDHA, which is upregulated in gynaecologic cancers, is important for cell proliferation and chemoresistance [74–76]. Rucaparib, a PARP inhibitor, suppresses the LDH-mediated conversion of pyruvic acid to lactic acid, contributing to pharmacological effects [77]. According to Xiang et al., inhibition of LDH-A can significantly boost the inhibitory effects of PARP inhibitors on OC with wild-type BRCA [78]. A small molecular inhibitor of LDHA named FX-11 was reported to induce reactive oxygen species in cancer cells with eventual cell death by perturbing the NADH/NAD + ratio [79]. Several additional LDHA inhibitors, such as oxamate, gossypol, galloflavin, and N-hydroxy indole-based inhibitors, have been explored in preclinical settings [79–85]. Although compounds that target lactate metabolism have not yet been approved, highly selective and efficient LDH inhibitors are an attractive cancer therapy.

#### PGI

Phosphoglucose isomerase (PGI) catalyses the interconversion of fructose 6 phosphate (F6P) and G6P during glycolysis. The autocrine motility factor (AMF) site, a conserved region, has been shown to be connected to the cytokine function of PGI. Through the phosphatidylinositol 3-kinase/protein kinase B (PI3K/AKT) pathway, PGI promotes growth and survival [86]. AMF/PGI secretion increases in EC in both tissues and serum. Meanwhile, MAPK-ERK signalling in EC, which is a potential therapeutic target, mediates carcinogenesis caused by AMF/PGI [87].

#### ALS

Fructose 1,6-bisphosphate (F1,6BP) is transformed by aldolases (ALS) into G3P and dihydroxyacetone phosphate (DHAP). It has three isoforms, aldolase A (ALDOA), aldolase B (ALDOB) and aldolase C (ALDOC) [86]. ALDOA is most related to gynaecologic cancers. In contrast to benign serous OC, Simonas et al. found that ALDOA was substantially expressed in cyst fluid and serum in OC Type 1 and Type 2 [87]. In uterine cervical cancer, ALDOA was substantially expressed, and this high expression facilitated EMT and activated HIF-1 to boost malignant potentials [88].

### GLUTs

GLUT1 has been implicated not only in physiological conditions but also in pathologic diseases ranging from inflammation to cancer as the most important carrier of mammalian glucose transport across cell membranes into the cell. GLUTs are overexpressed in most cancer cells to facilitate glucose consumption, especially in advanced and metastatic stages [89]. In several studies, GLUT1 overexpression has been linked to a poor prognosis in gynaecologic cancers [90, 91]. Overexpression of miR-1204 enhances GLUT-1 expression, glucose consumption, and cell proliferation, promoting the development of ovarian squamous cell carcinoma [92]. BA Y-876, a selective GLUT1 targeting inhibitor, adequately limited glycolytic metabolism and OC development in vitro and in vivo [93]. STF31 is a pyridylanilothiazole that inhibits glucose absorption and causes necrotic cell death in glycolytic cancer cells by interacting directly with the central pore of the GLUT1 transporter [94]. When STF31 was coupled with metformin, an inhibitor of oxidative OXPHOS, significant suppression of OC cell proliferation was observed [57]. Its other inhibitors, Ph, WZB117, and STF-31, also suppressed cell growth in other cancers [94–96].

### HIF-1α

The transcriptional activator HIF-1α is a key regulator of the Warburg effect. Under normal circumstances, the proline hydroxylase domain (PHD) protein hydroxylates HIF-1α, causing it to engage with the E3 ubiquitination ligase von Hippel‒Lindau (VHL). The interaction between HIF-1α and VHL causes HIF-1α to be degraded by the proteasome. Under hypoxia, on the other hand, the loss of disposal causes metabolic reprogramming by promoting glycolysis and inhibiting OXPHOS. The abundance of HIF-1α represses PDH while upregulating PDK. It also increases the expression of GLUT and other glycolytic enzymes [97–99]. The role of HIF-1α in gynaecologic cancer progression has been confirmed. A recent study showed that salt-inducible kinase 2 (SIK2) increased glucose metabolism reprogramming in OC via the PI3K/AKT/HIF-1α pathway [100]. Furthermore, Xiang et al. indicated that histone deacetylase inhibitors (HDACi) could suppress HIF-1α expression by inhibiting phosphatidylinositol 3-kinase (PI3K) and glycogen synthase kinase 3β (GSK3β) and enhance HIF-1α degradation by HSP70 independent of pVHL [101].

## The mode of action of lncRNAs in glycolysis

Studies have revealed that some glycolytic enzymes have tumoricidal effects in vitro and in vivo. When glycolytic inhibitors are used with chemotherapy, growth inhibition is considerably intensified. Unfortunately, considering the importance of glycolysis in normal cell glucose metabolism, there has been limited therapeutic success. Many studies have focused on glycolysis in gynaecologic cancers from the perspective of coding genes and proteins, but research progress has not been satisfactory. Through diverse molecular mechanisms, lncRNAs are currently thought to play key roles in tumour development [102, 103]. Upregulating glycolysis is one of the mechanisms of lncRNAs in cancer [104–107]. For example, lncRNA AGPG regulates PFKFB3-mediated tumour glycolytic reprogramming in oesophageal squamous cell carcinoma (ESCC) [25]. HISLA enhances aerobic glycolysis and apoptosis resistance in breast cancer cells [108].

**Fig. 1:**
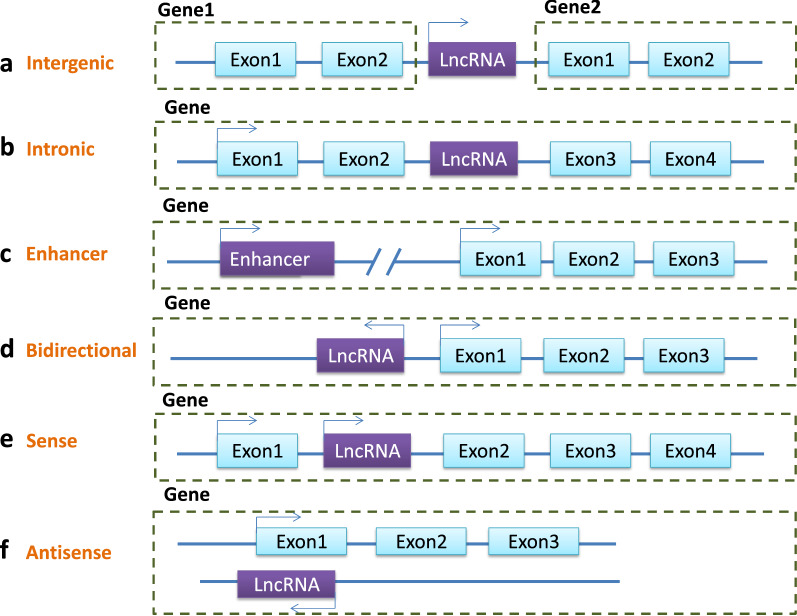
The six types of LncRNAs: **A** Intergenic lncRNAs are transcribed between two coding transcripts. **B** Intronic lncRNAs are expressed from the intron of a protein-coding gene. **C** Enhancer RNAs are transcribed from the enhancer. **D** Bidirectional lncRNAs are transcribed from the promoter of a protein-coding gene but in the opposite direction. **E**–**F** Sense lncRNAs or antisense lncRNAs are expressed from a protein-coding gene’s sense or antisense RNA strand

According to the origin of their expression, lncRNAs may be divided into six types: intergenic lncRNAs, intronic lncRNAs, enhancer RNA (eRNA), bidirectional lncRNAs, sense lncRNAs, and antisense lncRNAs [109–111] (Fig. 1).

**Fig. 2:**
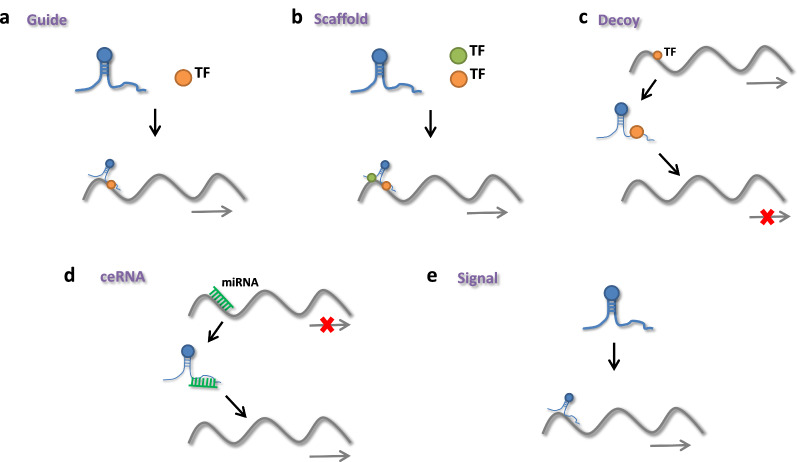
lncRNAs functions: **A** Guide: lncRNAs can attach to other molecules including protein, DNA, and RNA to direct them to specific genomic sites where they can control target gene expression. **B** Scaffold: lncRNAs can operate as a scaffold, allowing certain regulatory cofactors to join together to form a complex that activates target genes. **C** Decoy: lncRNAs can regulate gene expression by preventing transcriptional regulators from binding to their binding sites. **D** ceRNA: By interacting with miRNA and limiting its action, lncRNAs can behave as a microRNA sponge. **E** Signal: lncRNAs can control gene regulation by combining signaling pathways or transcription factors, or by directly interacting with DNA

Functionally, five major mechanisms for widely varying classes of lncRNAs have emerged [112–114] (Fig. 2): (1) Guide: lncRNAs may attach to other molecules to direct them to specific genomic locations where target gene expression is regulated. HOTAIR can cause chromatin-modifying enzymes such as polycomb repressive complex-2 (PRC2) to bind to target genes and control their expression [115]. Wang et al. found that lncRNA LINRIS promoted glycolysis via the LINRIS/IGF2BP2/MYC axis in colorectal cancer [116]. LncRNA CASC9 increased pancreatic cancer glycolysis and epithelial–mesenchymal transition (EMT) by binding to protein kinase B (AKT) to actively regulate HIF-1 signalling [117]. The lncRNA GLCC1/HSP90/c-Myc/LDHA axis promotes colorectal carcinogenesis and glucose metabolism [118]. SNHG6 enhances glycolysis via the SNHG6/hnRNPA1/PKM axis in colorectal cancer (CRC) [119]. LncSLCC1 can attach to AHR and actively control HK2 gene expression, inducing glycolysis activation and tumour development in CRC [120]. (2) Scaffold: lncRNAs can operate as scaffolds, allowing particular regulatory cofactors to join together to form a complex that activates target genes. The RBBP5 and GCN5 complexes were recruited to the PFKFB3 promoter using LINC00930 as a scaffold. It promoted glycolytic flux and cell cycle progression in nasopharyngeal cancer by increasing H3K4 trimethylation and H3K9 acetylation levels in the PFKFB3 promoter region, which epigenetically transactivates PFKFB3 [121]. (3) Decoy: lncRNAs can regulate gene expression by preventing transcriptional regulators from binding to their binding sites. (4) ceRNA: LncRNAs can act as sponges to titrate microRNAs (miRNAs) away from their mRNA targets, reducing target gene suppression. The overexpression of TUG1 promoted cell metastasis and glycolysis via the TUG1/miR-455-3p axis in hepatocellular carcinoma [122]. Through the miR-199a-5p/c-Myc axis, LINC01123 enhances non-small cell lung cancer proliferation and aerobic glycolysis [123]. In colorectal cancer, lncRNA MIR17HG promotes glycolysis by upregulating HK1 transcription by sponging miR-138-5p. By interacting with miR-222-3p and activating the HIPK2/ERK/c-myc pathway [124]. LINC00261 increases aerobic glycolysis. In pancreatic cancer, LINC00261 could also lower c-myc expression by sequestering IGF2BP1 [125]. (5) Signalling: LncRNAs control gene regulation by interacting directly with DNA or collaborating with signalling pathways or transcription factors. Wang et al. revealed that lncRNA HULC promoted glycolysis by directly binding to LDHA and PKM2 in liver cancer [126]. Liu et al. revealed that AGPG regulated PFKFB3 to reprogram glycolysis in oesophageal squamous cell carcinoma (ESCC) [25]. Through a hypoxia responsive element (HRE) motif, lnc-Dpf3 binds to HIF1a and suppresses its function [127]. LncRNA FEZF1-AS1 may bind to and stabilize PKM2, increasing aerobic glycolysis in CRC [128]. SNHG6 enhances glycolysis via the SNHG6/hnRNPA1/PKM axis in CRC cells [119].

As a result, lncRNA-mediated glucose metabolism might occur via four possible mechanisms: (1) altering glycolytic enzyme expression levels, (2) altering the expression levels and distribution of GLUTs, or (3) regulating glycolysis-related transcription factors or signalling pathways [129–131]. These findings show that lncRNAs operate as upstream regulators of glucose metabolism and might be utilized to discover new therapeutic targets for cancer prevention.

## LncRNAs are key regulators of glycolysis in gynaecologic cancers

### CeRNA

#### HOXB-AS3

**Fig. 3:**
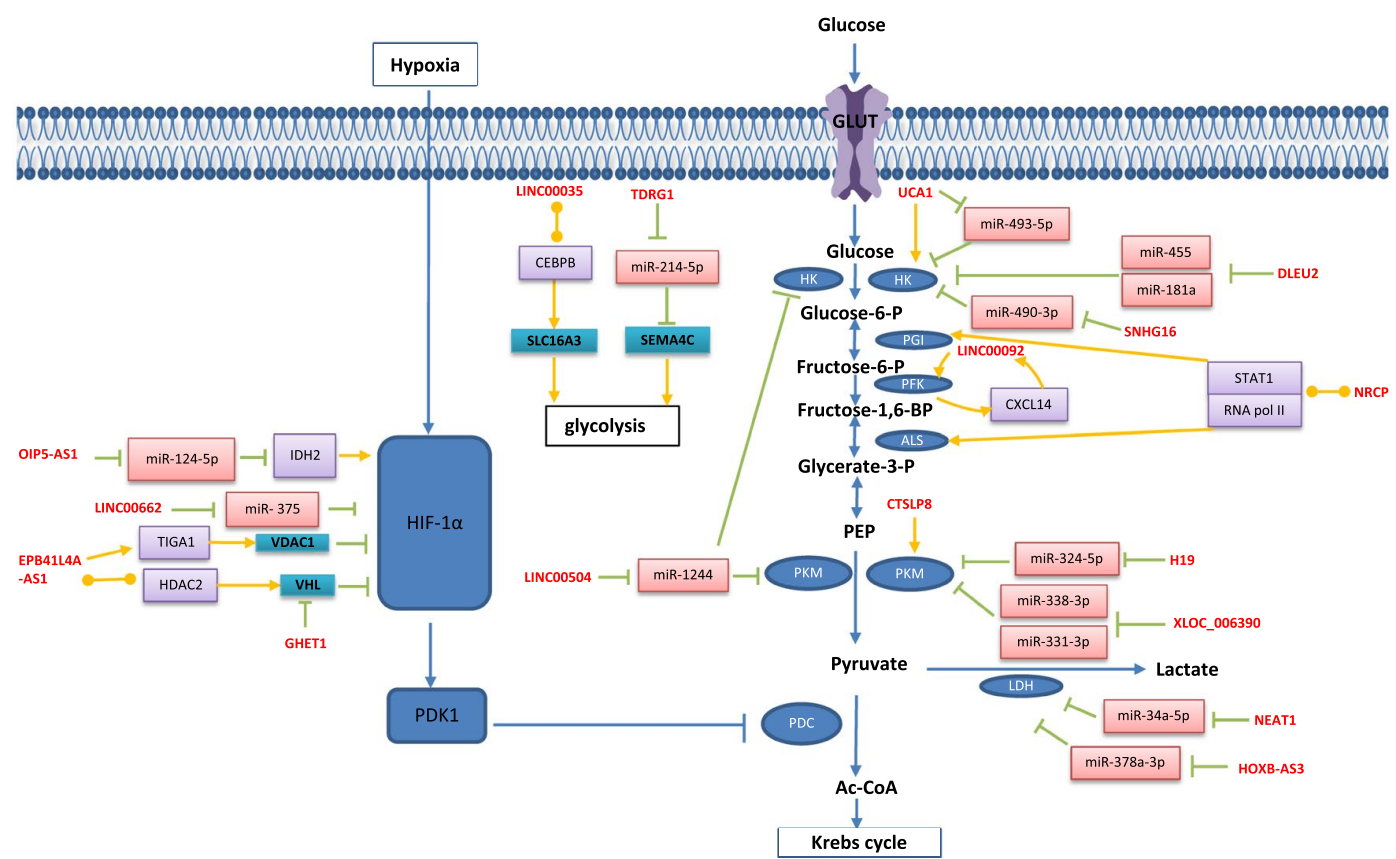
LncRNAs regulate glycolysis in gynecologic cancers: HOXB-AS3, LINC00504, SNHG16, DLEU2, H19, NEAT1, TDRG1, XLOC_006390, OIP5-AS1, UCA1, LINC00662 act on downstream target genes by the mechanism of ceRNA, UCA1, CTSLP8, LINC00092, EPB41L4A-AS1 act on downstream target genes by the mechanism of signal, EPB41L4A-AS1, LINC00035 act on downstream target genes by the mechanism of guide, NRCP acts on downstream target genes by the mechanism of scaffold, GHET1 acts on downstream target genes by the mechanism of decoy

HOXB-AS3 was found in both the nucleus and the cytoplasm, with a higher concentration in the latter. Xu et al. discovered that HOXB-AS3 was upregulated in OC tissue and was adversely correlated with the prognosis of OC patients. Furthermore, when HOXB-AS3 sponges miR-378a-3p, LDHA, which is a target of miR-378a-3p, may be upregulated to modulate the Warburg effect in OC cells. Moreover, the HOXB-AS3/miR-378a-3p/LDHA axis may enhance OC growth, invasion, and migration and hence serve as an independent prognostic marker for OC patients [132]. However, Huang et al. found that HOXB-AS3 was a tumour suppressor as a small peptide rather than a lncRNA by competitively binding to the arginine residues in an RNA-binding RGG box motif of hnRNP A1, which could bind to the sequences flanking PKM exon 9 to promote PKM2 formation, suppressing cancer growth as well as glycolysis in colon cancer [133] (Fig. 3, Table 1).

**Table 1:** Selected lncRNAs involved in glycolysis of gynecologic cancers

Mechanism	LncRNAs	Molecules and signaling pathways involved	Expression	Phenotypes affected	Cancer	Refs.
ceRNA	HOXB-AS3	HOXB-AS3/miR-378a-3p/LDHA axis	Up	Proliferation ( +) Migration ( +) Invasion ( +)	OC	[132]
	LINC00504	LINC00504/miR-1244/PKM2, HK2, PDK1 axis	Up	Proliferation ( +) Apoptosis (-)	OC	[136]
	SNHG16	TFAP2A/SNHG16/miR-490-3p/HK2 axis	Up	Proliferation ( +)	EC	[152]
	DLEU2	(1) DLEU2/miR-455/HK2 axis (2) EZH2/DLEU2/miR-181a/HK2 axis	Up	EMT ( +)	EC	[155]
	H19	H19/miR-324-5p/PKM2 axis	Up	Apoptosis (−) Proliferation ( +) Migration ( +) Invasion ( +)	OC	[146]
	NEAT1	NEAT1/miR-34a-5p/LDHA axis	Up	5-Fu resistance ( +) Proliferation ( +) Migration ( +)	CC	[158]
	TDRG1	TDRG1/miR-214-5p/SEMA4C axis	Up	Invasion ( +) Proliferation ( +)	CC	[163]
	XLOC_006390	XLOC_006390/miR-338-3p, miR-331-3p/PKM2 axis	Up	Migration ( +) Invasion ( +) Apoptosis (−) Metastasis ( +)	CC	[165]
	OIP5-AS1	miR-124-5p/IDH2/HIF-1α pathway	Up	Proliferation ( +)	CC	[168]
	UCA1	UCA1/miR-493-5p/HK2	Up	Proliferation ( +)	CC	[182]
	LINC00662	LINC00662/miR-375/HIF-1α axis	Up	Proliferation ( +)	OC	[172]
Signal	UCA1	UCA1/HK2 pathway	Up	Radio-resistance ( +)	CC	[183]
	CTSLP8	CTSLP8/PKM2 pathway	Up	Proliferation ( +) DDP resistance ( +)	OC	[177]
	LINC00092	CXCL14/LINC00092/PFKFB2 reciprocal feedback loop	Up	Migration ( +) Invasion ( +)	OC	[175]
Guide	EPB41L4A-AS1	(1) recruit HDAC2 into the VHL inducing HIF-1αaccumulation (2) EPB41L4A-AS1/TIGA1/VDAC1/HIF-1α pathway	Down	Proliferation (−) Apoptosis ( +) Migration (−) Invasion (−) Cisplatin-resistance (−)	CC	[184]
	LINC00035	recruit CEBPB into the SLC16A3 promoter region, increasing the SLC16A3 transcription	Up	Viability ( +) Apoptosis (−) Migration ( +) Invasion ( +)	OC	[190]
Scaffold	NRCP	as a Scaffold between STAT1 and RNA polymerase II, leading to increased expression of GPI, ALDOA, and ALDOC	Up	Proliferation ( +) Apoptosis (−)	OC	[192]
Decoy	GHET1	GHET1/HIF-1αpathway	Up	Proliferation ( +)	OC	[195]

#### LINC00504

LINC00504 was shown to have a role in cancer cell proliferation, invasion, and metabolism by acting as a ceRNA in breast cancer and lung cancer [134, 135]. Furthermore, by serving as an endogenous sponge for miR-1244, LINC00504 decreased miR-1244 expression, increased PKM2, HK2, and pyruvate dehydrogenase kinase 1 (PDK1) expression, and promoted glycolysis in OC cells and vice versa [136].

#### H19

H19 is one of the first lncRNAs to be discovered, and it is encoded via the H19 locus on chr11p15.5 [137]. Before polyadenylation, the H19 gene is translated into a lncRNA by the RNAPII enzyme, acquiring a 5' cap and spliced into 2.3-kb RNA. H19 is both paternally and maternally imprinted [138]. Except for certain organs, such as the uterus and breast, the expression of H19 is suppressed after the embryo's full development and delivery [139].

H19 overexpression has been linked to OC [140]. Ginsenoside 20(S)-Rg3, an active saponin monomer derived from red ginseng, was found to have antitumour activity [141–143]. Moreover, prior research has revealed that ginsenoside 20 (S)-Rg3 antagonizes the Warburg effect in OC and inhibits HK2 and PKM2 [144, 145]. Zheng et al. found that H19 was one of the most decreased lncRNAs in 20 (S)-Rg3-treated SKOV3 cells and identified PKM2-targeting miR-324-5p as the target miRNA sponged by H19. As a result, they indicated that the H19/miR-324-5p/PKM2 pathway was the source of 20 (S)-Rg3’s anti-Warburg activity [146].

To harness H19 overexpression for targeted treatment in OC, an open-label, dose-escalation phase 1/2a clinical study was conducted. By using Intraperitoneal DTA-H19 (BC-819), a DNA plasmid that encodes the A portion of diphtheria toxin (DTA) regulated by the promoter sequence of the H19 gene promoter, tumour size decreased by 40%. Overall survival (OS) rates ranged from 6.3 to 15.0 months. As a result, the researchers found that patients tolerated DTA-H19 well and that the greatest response was shown with steady ascites and tumour size. This therapy may be more useful for ovarian/peritoneal tumours that are less advanced [147].

#### SNHG16

SNHG16 is an important tumour-associated lncRNA that regulates many miRNAs, including miR-216-5p [148], miR-455-3p [149], and miR-605-3p [150]. A 7571-bp region encodes it on chromosome 17q25.1. In certain studies, SNHG16 expression increased in EC tissue and cells. The poor survival rate and recurrence-free survival of EC patients were linked to ectopic SNHG16 overexpression [151]. Zhang et al. demonstrated that SNHG16 plays an important role in EC proliferation and glycolysis via the TFAP2A/SNHG16/miR-490-3p/HK2 axis. The transcription factor TFAP2A bound to the promoter region of SNHG16 and activated its transcription upstream. The suggested mechanism of action was that SNHG16 competitively sponged miR-490-3p, which led to HK2 upregulation and consequently promoted glycolysis, offering interesting insight into EC tumorigenesis [152].

#### DLEU2

DLEU2 is found on chromosome 13q14.2 [153]. Human cancer cell lines express DLEU2 in their cytoplasm and nucleus [153]. By targeting miR-128-3p, DLEU2 knockdown decreased cell proliferation, caused apoptosis and cell cycle arrest at the G2/M phase of CC cells in vitro, and suppressed tumour development in vivo [154]. According to Dong et al. DLEU2 was expressed at greater levels in stage 1 EC tissues than in normal endometrial tissues. Furthermore, elevated DLEU2 expression was linked to a lower overall survival rate. DLEU2 also controlled the HK2/FAK/ERK1/2 axis by binding competitively to miR-455 and interacting with EZH2 to silence miR-181a expression, enhancing glycolysis, EMT [155].

#### NEAT1

As a result of alternative 30-end processing, NEAT1 is transcribed from a particular location on human chromosome 11 by RNAPII to create two different isoforms, NEAT1-1 (3.7 kb) and NEAT1-2 (22.7 kb) [156]. In the same cancer type, different NEAT1 isoforms may have different roles. Knocking down NEAT1-1 inhibited cell invasion and proliferation in colorectal cancer cell lines but knocking down NEAT1-2 boosted cell growth [157]. As a result, future research should focus on determining the particular processes through which NEAT1 isoforms cause human tumours to develop. Nuclear-rich NEAT1 was found to be highly upregulated in CC, particularly in 5-Fu resistant CC; according to Shao et al., NEAT1 functioned as a ceRNA of miR-34a in CC cells, decreasing the cellular glycolysis rate and sensitizing 5-Fu resistant cells by upregulating the expression of LDHA, a glycolysis-important enzyme [158]. Future research into the aforementioned molecular route in an in vivo animal model is ongoing.

#### TDRG1

Previous studies have shown that TDRG1 regulates cancer progression by regulating multiple miRNAs, including miR-873-5p [159] and miR-326 [160]. TDRG1 was shown to be significantly expressed in CC tissues and cells, and its expression was upregulated in response to hypoxia [160–162]. Using online databases and functional studies, Li et al. established that TDRG1 controlled semaphorin 4C (SEMA4C) expression in CC by acting as a sponge of miR-214-5p. By boosting glucose absorption and lactate production, TDRG1 promotes invasion and glycolysis in CC cells [163]. However, the mechanism of SEMA4C in glycolysis remains a mystery.

#### XLOC_006390

XLOC 006390 is an intergenic lncRNA that is 4,899 nucleotides in length. It has been shown to increase pancreatic cancer cell proliferation and migration through the XLOC 006390/c-Myc/GDH1 signalling pathway [164]. Luan et al. found overexpression of XLOC 006390 in CC cells. They also found that silencing XLOC 006390 increased miR-331-3p and miR-338-3p levels. Moreover, the XLOC 006390/miR-338-3p, miR-331-3p/PKM2 axis has been shown to increase CC cell migration, glycolysis, and invasion while inhibiting cell apoptosis [165]. However, the systematic mechanism of XLOC 006390 needs to be explored in the future.

#### OIP5-AS1

OIP5-AS1, a newly discovered and promising lncRNA, was found on chromosome 15q15.1 [166]. According to a previous study, high levels of OIP5AS1 were associated with a poor prognosis for CC patients and enhanced the proliferation of CC cells in vitro and in vivo [167]. Li et al. reported that OIP5AS1 inhibited miR-124-5p expression levels by increasing IDH2 expression. IDH2 increased CC glycolysis by upregulating HIF-1α [168]. IDH2, an isocitrate dehydrogenase, is a crucial protein in the tricarboxylic acid cycle in mitochondria. The regulation of HIF-1α expression by mitochondrial NADPþ-dependent isocitrate dehydrogenase (IDPm) is mediated through the PI3K/Akt pathway in prostate cancer [169]. However, the underlying mechanistic details about how IDH2 regulates HIF-1α remain unclear.

#### LINC00662

LINC00662, encoded by a 7571-bp region on chromosome 17q25.1, is an oncogenic lncRNA that functions primarily as a ceRNA to regulate several miRNAs, such as miR-199a-5p [170] and miR-16-5p [171]. LINC00662 was found to be strongly expressed in OC tissues and cells by Tao et al., and patients with greater LINC00662 expression had a poor prognosis. They also revealed that LINC00662 served as a sponge for miR-375, promoting HIF-1 production and OC cell proliferation and glycolysis [172].

### Signal

#### LINC00092

LINC00092 is a gene with no functional annotations and is found in the intergenic regions of 9q22.32. Linc00092 has been found as a glycolysis-related lncRNA in breast cancer cells and cardiac fibroblast [173, 174]. Zhao and his colleagues discovered that LINC00092 functions in OC cell proliferation and metastasis via cancer-associated fibroblasts (CAFs). LINC00092 was shown to be a downstream effector in CXCL14-high CAF-promoted OC development; by interacting directly with PFKFB2, LINC00092 stimulated the production of PFKFB2. F2,6BP might activate PFK-1 and accelerate glycolysis. Moreover, preserving CAF-like fibroblast features required the PFKFB2-induced glycolytic phenotype of OC cells, implicating a reciprocal feedback loop between CXCL14-positive CAFs and OC cells [175].

#### CTSLP8

CTSLP8 was discovered in OC, and its expression in metastatic tumour tissues was considerably higher than that in initial ovarian tumours. CTSLP8 promoted the proliferation, migration, EMT, and invasion of OC cells through upregulation of CTSL1 by functioning as a ceRNA against miR-199a-5p [176]. Li et al. verified that the CTSLP8/PKM2 complex upregulates c-Myc expression by interacting with the promoter region, resulting in the overexpression of GLUT1 and LDHA [177].

#### UCA1

UCA1 was first found and studied in the context of bladder cancer [178]. UCA1 acts as an oncogenic lncRNA in several different cancers [179]. Through the UCA1/miR-125a/HK2 axis, UCA1 knockdown decreased chemoresistance in acute myeloid leukaemia by reducing glycolysis [180]. In oesophageal cancer, UCA1 can promote tumour glucose metabolism via the UCA1/miR-203/HK2 axis, contributing to cancer cell proliferation and metastasis [181]. In CC, UCA1 can promote glycolysis by the UCA1/miR-493-5p/HK2 axis, contributing to CC development [182]. Radiation increased the expression of UCA1, according to Li et al. UCA1 increased glycolysis by directly targeting the key enzyme HK2, which influences CC cell sensitivity [183]. However, further study is needed to understand the mechanism underlying the regulatory effects of the UCA1/HK2/glycolysis pathway on radio-resistance.

### Guide

#### EPB41L4A-AS1

EPB41L4A-AS1 is found in the 5q22.2 region of the genome, which has a close correlation to cancer due to frequent DNA fragment deletion. Low expression and deletion of EPB41L4A-AS1 have been linked to poor prognosis in cancer patients, including those with CC. EPB41L4A-AS1 is a p53-independent gene. In the nucleolus, EPB41L4A-AS1 interacted and colocalized with HDAC2 and NPM1, according to Liao et al. Knockdown of EPB41L4A-AS1 decreased the interaction between HDAC2 and NPM1, resulting in HDAC2 translocation from the nucleolus to the nucleoplasm, where it interacted with the promoters of VHL, VDAC1, and other unidentified target genes to reduce gene transcription by reducing H3K27 occupancy on promoter regions [184]. HIF-1α accumulation can be induced by various methods. On the one hand, knocking down EPB41L4A-AS1 increased HIF-1α expression by decreasing VHL expression. On the other hand, VDAC closure or reduction decreases metabolite exchange. It enhances intramitochondrial oxidative stress by inhibiting O2 efflux from the IMS to the cytosol, increasing cellular stress and ROS. It can activate p38 MAPK and cause HIF-1α accumulation [185, 186], as well as stabilize HIF-1α by inactivating prolyl hydroxylases (PHDs) and activating the initiation factor 2 (P-eIF2a)/activating transcription factor 4 (ATF4) pathway [186–188]. According to Yabuta et al., the EPB41L4A-AS1 gene also encodes TIGA1, a small mitochondrion-associated protein. In soft agar, its ectopic expression reduced tumour cell colony formation and tumour development [189]. In addition, similar to VDAC, TIGA1 interacted with a-tubulin. Microtubules were destabilized by TIGA1 knockdown, which resulted in an increase in free a-tubulin and its binding to VDAC1, causing the VDAC channel to be partially blocked. After inhibiting VDAC, HIF-1α accumulation and the P-eIF2a/ATF4 pathway were activated. Finally, knocking down EPB41L4A-AS1 boosted glycolysis via the HIF-1α pathway and increased glutamine metabolism via the P-eIF2a/ATF4 pathway [188]. However, the mechanisms that control the shuttling of chromatin regulators between the nucleoplasm and nucleolus remain unexplained. Furthermore, it is unknown how chromatin regulators are recruited to the right chromatin locations.

#### LINC00035

Yang et al. discovered that LINC00035 was activated in OC and was able to attract the transcription factor CEBPB to the SLC16A3 promoter region, boosting SLC16A3 transcription. SLC16A3 overexpression repressed OC cell viability, migration, invasion, and glycolysis and reduced apoptosis [190]. SLC16A3, also known as monocarboxylate transporter 4 (MCT4), is involved in lactate export during glycolysis. MCTs have a molecular chaperone called CD147. Wang et al. showed that lysine methyltransferase 5A (KMT5A) dimethylated CD147 to CD147-K234me2, which boosted MCT4 membrane translocation through direct interactions in non-small cell lung cancer cells, resulting in increased glycolysis and lactate export [191]. However, further research regarding how CD147 affects MCT4 in gynaecologic cancers is needed.

### Scaffold

#### NRCP

According to one study, NRCP can act as a scaffold between STAT1 and RNA pol II, resulting in enhanced expression of downstream target genes such as GPI, ALDOA and ALDOC. The involvement of NRCP in metabolic changes in OC cells was discovered by Rupaimoole et al. They discovered that NRCP was the most elevated lncRNA in OC and that silencing NRCP deceased glycolysis and boosted mitochondrial respiration in cancer cells. They also confirmed the substantial decrease in initial tumour development and metastasis following nanoliposome-delivered si-NRCP to tumours using orthotopic OC animal models [192].

### Decoy

#### GHET1

GHET1 has a length of 1913 bp and is found on chromosome 7q36.1 [193]. Knockdown of GHET1 has been shown to inhibit CC proliferation, migration, and invasion [194]. According to Liu et al., GHET1 overexpression was detected in OC and was linked to a worse outcome in cancer patients. GHET1 knockdown inhibited OC cell growth and induced apoptosis. GHET1 interacted with VHL and prevented VHL-mediated HIF-1 degradation, increasing HIF-1 protein levels in OC cells. Upregulation of HIF-1 increased glucose absorption and lactate production in OC cells by upregulating glycolysis [195].

## Discussion

For gynaecologic cancers with high drug resistance, proliferation, migration, invasion, metastasis, and EMT, it is crucial to investigate regulation of the glycolytic pathway. Cancer cell fate is strongly correlated with energy metabolism. A low pH cancer microenvironment may be maintained, and the energy required for cancer cell growth can be supported by glycolysis. Additionally, a significant amount of nucleic acid precursors can be created to facilitate cell proliferation. Unfortunately, considering the importance of glycolysis in normal cell glucose metabolism, there has been limited therapeutic success.

LncRNAs are novel players in cancer glycolysis, and studying metabolism-related lncRNAs might help researchers better understand the regulatory mechanisms governing gynaecologic cancers' biological processes. Despite the use of targeted medications such as PARP inhibitors, the treatment of gynaecologic tumours is still in its initial stages, owing to resistance to chemotherapy, which results in a considerable reduction in the survival rate of patients with OC. The lncRNA-based approach to glycolysis has various advantages. One of them is that, in contrast to targeting glycolytic enzymes, targeting a single lncRNA directly may have no effect on normal cells. Similarly, the expression of certain proteins that are considered non-targetable can be manipulated by targeting a specific lncRNA. Because many lncRNAs are very specific, they can remain stable in body fluids. Furthermore, the evaluation of lncRNA levels is extremely promising in diagnosis and prognosis.

However, the molecular mechanisms and roles of the great majority of lncRNAs remain unknown. There are still many challenges. First, the specific mechanism by which lncRNAs act on glycolysis in gynaecologic cancers is unclear. The mechanism of lncRNAs acting on downstream target genes is similar to that of ceRNAs. Guides, scaffolds, decoys, and signals are rare and still need to be explored. Many studies have found that m6A regulatory factors can regulate the expression of enzymes related to the glucose metabolism pathway, thus affecting glycolysis in tumours [196, 197]. Ferroptosis, immunosuppression and immune escape are also related to tumour cell metabolic reprogramming. These findings provide a reference for us to study the specific mechanism of lncRNAs acting on glycolysis in gynaecologic cancers. Second, the upstream mechanism of lncRNA is also not precise. Last, no specific lncRNA has been found in gynaecologic cancers. Many studies have examined particular markers of gynaecologic cancers [198, 199], but the effect is minimal. Typically, the target proteins changed in gynaecologic malignancies do not have mutations in their coding sequence. Because of the lack of DNA changes, direct sequencing of the DNA of these genes may result in erroneous molecular diagnosis. Indeed, lncRNA dysregulation occurs mostly at the post-transcriptional level, leaving the DNA untouched. As a result, the molecular diversity in various patients is greatly underestimated, potentially leading to ineffective therapy. Most lncRNAs that have been identified thus far influence just a small number of cell processes that are critical for cell replication and survival. These findings imply that effective methods of impairing their function may be promising treatment options for gynaecologic cancers as well. The disadvantage is that many targets are targeted at the same time, either directly or indirectly, amplifying the impact of the dysregulated lncRNA inside cancer cells. Because certain metabolic pathways are redundantly changed, concentrating on only one target protein may not be enough to treat gynaecologic cancers efficiently. Precision medicine in cancer is based on therapeutic pathways that are personalized to patients’ genetic and epigenetic characteristics. The diagnostic and predictive significance of lncRNAs can be improved by improving classification methods based on genetic and epigenetic events in gynaecologic cancers. This has been demonstrated in EC [198, 200].

There is currently no FDA-approved lncRNA-based therapeutic available. The specific characteristics will be a milestone for the diagnosis and treatment of gynaecologic cancers if we can obtain specific results.

## Conclusion

Based on the current predictive ability of biomarkers, we need to explore more accurate and consistent biomarkers and other types of lncRNA screening methods in the future. LncRNAs play an essential role in drug resistance and prognosis in gynaecologic cancers by regulating glycolysis, so targeted treatment of lncRNAs presents a potential hope of a radical cure. In general, the link between lncRNAs and glycolysis is interesting and urge us to explore further, which will contribute to targeted therapy and new combination therapies and lead to long-term benefits for patients.

## Data Availability

All data generated or analyzed during this study are included in this published article and its supplementary information files.

## Abbreviations

OC: Ovarian cancer
CC: Cervical cancer
EC: Endometrial cancer
lncRNAs: Long non-coding RNAs
HIF-1α: Hypoxia-inducible factor-1α
OXPHOS: Mitochondrial oxidative phosphorylation
PET: Positron emission tomography
PPP: The pentose phosphate pathway
GSH: Glutathione
TKTL1: Transketolase
G6P: Glucose-6phosphate
VDACs: Voltage-dependent anion channels
2-DG: 2-Deoxyglucose
3-BrPA: 3-Bromopyruvate
F2,6BP: Fructose-2,6-bisphosphate
ROS: Reactive oxygen species
PHD: Proline hydroxylase domain
VHL: Von Hippel-Lindau
HDACi: Histone deacetylase inhibitors
eRNA: Enhancer RNA
ESCC: Esophageal squamous cell carcinoma
CRC: Colorectal cancer
OS: Overall survival
DTA: Diphtheria toxin
CAF: Cancer-associated fibroblasts
PHDs: Prolyl hydroxylases
PARP: Poly (ADP-ribose) polymerase
GLUTs: Glucose transporters
ATP: Adenosine triphosphate
NADPH: Nicotinamide adenine dinucleotide
HK: Hexokinase
3PO: Phosphofructokinase (PFK) 3-(3-pyridinyl)-1-(4-pyridinyl)-2-propen-1-one
PKM2: Pyruvate kinase muscle isozyme 2
mTOR: Mammalian target of rapamycin
LDHA: Lactate dehydrogenase A
PGI: Phosphoglucose isomerase
F6P: Fructose 6 phosphate
AMF: Autocrine motility factor
PI3K/AKT: Phosphatidylinositol 3-kinase/protein kinase B
ALS: Aldolases
F1,6BP: Fructose 1,6-bisphosphate
DHAP: Dihydroxyacetone phosphate
ALDOA: Aldolase A
ALDOB: Aldolase B
ALDOC: Aldolase C
SIK2: Salt-inducible kinase 2
PI3K: Phosphatidylinositol 3-kinase
GSK3β: Glycogen synthase kinase 3β
PRC2: Polycomb repressive complex-2
EMT: Epithelial–mesenchymal transition
AKT: Protein kinase B
miRNAs: MicroRNAs
HRE: Hypoxia responsive element
PDK1: Pyruvate dehydrogenase kinase 1
SEMA4C: Semaphorin 4C
IDPm: Itochondrial NADPþ-dependent isocitrate dehydrogenase
P-eIF2a: Initiation factor 2
ATF4: Activating transcription factor 4
MCT4: Monocarboxylate transporter 4
KMT5A: Lysine methyltransferase 5A
